# Cysteinyl leukotriene receptor-1 as a potential target for host-directed therapy during chronic schistosomiasis in murine model

**DOI:** 10.3389/fimmu.2024.1279043

**Published:** 2024-05-22

**Authors:** Paballo Mosala, Thabo Mpotje, Nada Abdel Aziz, Hlumani Ndlovu, Fungai Musaigwa, Justin Komguep Nono, Frank Brombacher

**Affiliations:** ^1^ Institute of Infectious Diseases and Molecular Medicine (IDM), Department of Pathology, Division of Immunology and South African Medical Research Council (SAMRC) Immunology of Infectious Diseases, Faculty of Health Sciences, University of Cape Town, Cape Town, South Africa; ^2^ International Centre for Genetic Engineering and Biotechnology (ICGEB), Cape Town Component, Cape Town, South Africa; ^3^ Immuno-Biotechnology Lab, Biotechnology Department, Faculty of Science, Cairo University, Giza, Egypt; ^4^ Division of Chemical and System Biology, Department of Integrative Biomedical Sciences, Faculty of Health Sciences, University of Cape Town, Cape Town, South Africa; ^5^ Unit of Immunobiology and Helminth Infections, Laboratory of Molecular Biology and Biotechnology, Institute of Medical Research and Medicinal Plant Studies (IMPM), Ministry of Scientific Research and Innovation, Yaoundé, Cameroon; ^6^ Welcome Centre for Infectious Diseases Research in Africa (CIDRI-Africa) and Institute of Infectious Disease and Molecular Medicine (IDM), Faculty of Health Sciences, University of Cape Town, Cape Town, South Africa

**Keywords:** schistosomiasis, montelukast, praziquantel, Th2 immune responses, cysteinyl leukotriene 1 (CYSLTR1), host-directed therapy

## Abstract

Schistosomiasis remains the most devastating neglected tropical disease, affecting over 240 million people world-wide. The disease is caused by the eggs laid by mature female worms that are trapped in host’s tissues, resulting in chronic Th2 driven fibrogranulmatous pathology. Although the disease can be treated with a relatively inexpensive drug, praziquantel (PZQ), re-infections remain a major problem in endemic areas. There is a need for new therapeutic drugs and alternative drug treatments for schistosomiasis. The current study hypothesized that cysteinyl leukotrienes (cysLTs) could mediate fibroproliferative pathology during schistosomiasis. Cysteinyl leukotrienes (cysLTs) are potent lipid mediators that are known to be key players in inflammatory diseases, such as asthma and allergic rhinitis. The present study aimed to investigate the role of cysLTR1 during experimental acute and chronic schistosomiasis using cysLTR1^-/-^ mice, as well as the use of cysLTR1 inhibitor (Montelukast) to assess immune responses during chronic *Schistosoma mansoni* infection. Mice deficient of cysLTR1 and littermate control mice were infected with either high or low dose of *Schistosoma mansoni* to achieve chronic or acute schistosomiasis, respectively. Hepatic granulomatous inflammation, hepatic fibrosis and IL-4 production in the liver was significantly reduced in mice lacking cysLTR1 during chronic schistosomiasis, while reduced liver pathology was observed during acute schistosomiasis. Pharmacological blockade of cysLTR1 using montelukast in combination with PZQ reduced hepatic inflammation and parasite egg burden in chronically infected mice. Combination therapy led to the expansion of Tregs in chronically infected mice. We show that the disruption of cysLTR1 is dispensable for host survival during schistosomiasis, suggesting an important role cysLTR1 may play during early immunity against schistosomiasis. Our findings revealed that the combination of montelukast and PZQ could be a potential prophylactic treatment for chronic schistosomiasis by reducing fibrogranulomatous pathology in mice. In conclusion, the present study demonstrated that cysLTR1 is a potential target for host-directed therapy to ameliorate fibrogranulomatous pathology in the liver during chronic and acute schistosomiasis in mice.

## Introduction

Cysteinyl leukotrienes (cysLTs) are a class of leukotrienes that include leukotriene C_4_ (LTC_4_), LTD_4_ and LTE_4_, all of which contain the amino acid cysteine conjugated to the lipid backbone (1). Cysteinyl leukotrienes are synthesized from arachidonic acid metabolism (2–4) that is released from the plasma membrane through the action cytosolic phospholipase A_2_. They are then synthesized to leukotriene A_4_ (LTA_4_) by 5-lipoxygenase acting with the assistance of 5-lipoxygenase activating protein. LTA4 is hydrolysed to either LTB_4_ through the action of LTA_4_ hydrolase or LTC_4_ by the action of LTC_4_ synthase. Moreover, LTC_4_ can be further processed into LTD_4_ and LTE_4_ by gamma glutamyl transferase (5). CysLTs act through two structurally divergent G-protein-coupled receptors, named cysteinyl leukotriene receptor-1 (cysLTR1) and cysLTR2 (6), which have been cloned for the human (7–11) and the mouse (12–15). CysLTR1 is thought to be the primary receptor mediating smooth muscle contraction and inflammatory cytokine production following exposure to antigen (16). Although the two receptors induce many of the same immune responses, cysLTR2 requires up to 10-fold concentration of LTD_4_, which is the most potent ligand for activation of cysLTs (17). The targeted inhibition of cysLTR1 with montelukast has been shown to offer some therapeutic benefits for the treatment of asthma and allergic rhinitis by blocking the secretion of Th2 cytokines and impairing airway inflammation (18).

CysLTs are produced by a variety of innate immune cells such as basophils (19), eosinophils (20), mast cells (21) and monocytes following exposure to allergens (22). In addition, cysLTs are generated following exposure to allergens as the product of IgE cross-linking and activation mast cells (1). Cysteinyl leukotrienes are potent inflammatory lipid mediators and have been reported to drive Th2 immunity (23, 24). Initiation and amplification of robust Th2 immune responses is crucial for conferring protective immunity to helminth infections in mice (25).

Similar to allergic responses, infection with helminthic parasites such as *Schistosoma* spp. induces robust type 2 immunity that is crucial for the induction of protective immune responses to helminth infections in humans and mice. However, the triggers of type 2 immune responses during schistosomiasis are poorly understood. *Schistosoma mansoni* (*S. mansoni*) triggers granulomatous inflammation that is induced by the eggs that are lodged in the tissues such as the liver in the case of *S. mansoni* infection or bladder in the case of *S. japonicum*. During the early stages of infection, the predominant immune response is characterized by Th1 immune responses targeted at adult worms. However, the immune response switches from Th1 to Th2 when the mature worms start laying eggs in the mesenteric venules that migrate and get trapped in the host tissues (26). It is crucial that there is efficient switching between Th1 to Th2 immunity to prevent lethal inflammatory pathology driven by uncontrolled Th1 (25, 27) and Th17 immunity. Finally, regulatory T cells emerges at week 10 of infection, which is regarded as the chronic phase of the disease (26, 28–30). The chronic phase of schistosomiasis causes excessive collagen deposition resulting in fibrosis (26, 31).

Liver fibrosis caused by schistosomiasis continues to pose a public health problem worldwide, and currently there are no drugs available for treatment and reversal of fibrosis (2). Beller et al. observed reduced inflammation and fibrosis in lungs of mice following long-term injury in the absence of cysLTR1. Furthermore, there was reduced lung inflammation in *S. mansoni* infected 5-lipoxygenase deficient (5-LOX^-/-)^ mice (32), suggesting that leukotrienes are essential in driving chronic inflammation in the lungs. However, it is unclear if cysLTR1 is required for driving liver fibrotic granulomatous inflammation during schistosomiasis. The present study investigates whether cysLTs are required for driving inflammation and fibrosis in the liver during chronic schistosomiasis.

Here, we investigated the role of cysLTs signalling through cysLTR1 in the development of fibrogranulomatous liver pathology during chronic schistosomiasis using *cysLTR1* gene deficient mice. We observed reduced hepatic fibrogranulomatous inflammation in the cysLTR1^-/-^ mice compared to littermate control mice during the chronic stage of schistosomiasis. Moreover, we found a reduced production of IL-4 in mice lacking cysLTR1 in comparison to the littermate control mice during chronic schistosomiasis. Remarkably, the inhibition of cysLTR1 with montelukast alone or in combination with praziquantel (PZQ) led to the reduction in fibrogranulomatous inflammation and liver egg burden compared to littermate control mice during chronic schistosomiasis. Although the disruption of cysLTR1 is dispensable for host survival during schistosomiasis, we observed significant weight loss during the acute stage of infection, indicating the crucial role cysLTR1 plays during acute schistosomiasis. Taken together, these findings demonstrated that interfering with the signalling of cysLTR1 using gene-deficient mice or small molecule inhibitors ameliorates fibrogranulomatous liver pathology during chronic and acute schistosomiasis.

## Methods

### Ethics statement

Ethical approval of mouse experiments was granted by the University of Cape Town (UCT) Health Sciences Animal Ethics Committee (Protocol number 016/027 and 020/002) in accordance with guidelines by the Animal Research Ethics Committee (AREC) of South African National Standard (SANS 10386:2008). All measures were taken to minimize the suffering of animal in accordance with the AREC guidelines.

### Mice

C57/BL6 background cysLTR1^-/-^ mice were received as a kind donation from Yoshihide Kanaoka at Harvard University. CysLTR1^-/-^ mice were backcrossed to BALB/c background for 10 generations for this study. Mice were bred and housed under specific pathogen-free conditions at the UCT animal facility, and mice aged between 8 and 12 weeks were used for the experiments.

### Infection of mice with *Schistosoma mansoni*

Mice were shaved and infected percutaneously with either 35 (chronic/late infection) or 100 (acute/early infection) live *S. mansoni* cercariae (33–36) shedded by infected *Biomphalaria glabrata* snails (NMRI strain, NR-21962, Biomedical Research Institute, Rockville, USA). Infected mice were monitored daily and weighed weekly in accordance with the AREC guidelines. Mice were euthanized using halothane and death was confirmed by cardiac puncture. Mice were killed at 8 weeks post-infection (acute infection) or 16 weeks post-infection (chronic infection).

### Preparation of montelukast

Aliquots of Montelukast soluble at 200 mg/ml in 100% EtOH were stored at -80°C. On day of treatment, the aliquots were thawed, and corn oil was added into the tube maintained in water bath and mixed before incubation for 1 hour. For 100μl corn oil preparation given at each oral gavage, 0.88mg of Montelukast was administered (35mg/kg). Treatment commenced at week 11 post infection, infected mice were treated once every second day, for three weeks.

### Preparation of praziquantel

The PZQ (Merck KGaA, Darmstadt, Germany) solution was prepared as previously described previously by Nono et al. (35). Briefly, PZQ was weighted and mixed with 10 parts 70% Tween and 30% Ethanol using a magnetic stirrer to achieve a concentration of 400mg/kg of animal body weight. Afterwards, 90 parts of sterile water was added to the solution while stirring with a magnetic stirrer until a homogenous solution was obtained. Each mouse was given 200μl of the homogeneous PZQ suspension by oral gavage within 2 h after it was prepared daily. At 11 weeks post infection, infected mice were treated once, daily for seven days.

### Histology

The harvested liver was fixed in buffered formalin (4% (v/v) formaldehyde in PBS), embedded in wax, and then processed. Sections were cut and stained with Haematoxylin and Eosin (H&E) to assess tissue pathology or stained with chromotrope analine blue solution (CAB) followed by counterstaining with Wegert’s haematoxylin for collagen detection. Images were captured using the Nikon Eclipse 90i light microscope (Nikon Corporation). Granuloma area was measured by computer-assisted morphological analysis using the NIS Elements Imaging software (Nikon Corporation) as previously discussed (33–36). We analysed an average of 20 granulomas per mouse which contained a single visible egg at the centre.

### Enzyme-linked immunosorbent assay

#### Serum antibody titres

Antigen-specific serum antibody isotypes (IgG1, IgG2a, IgG2b) and total IgE titres were determined from the plasma that was obtained from infected mice (37). Briefly, blood was collected by cardiac puncture and placed in serum separator tubes (BD Bioscience, San Diego, CA). The tubes were spun at 8 000×g for 10 minutes at 4°Cand the top layer constituting the plasma was collected. The Nunc MicroWell flat-bottom 96-well plates (Thermo Fisher Scientific) were coated with 10μg/ml soluble egg antigen (SEA), blocked with 2% (m/v) BSA for 3 hours at 37°C, the serum was loaded, and the plates were incubated overnight at 4°C. The plates were washed, and alkaline phosphatase-labelled secondary antibody was added and incubated for 2 hours at 37°C. The plates were washed, the substrate (4-nitrophenyl substrate, Sigma-Aldrich, St. Louis, Missouri), and absorbance was read at 405nm using VersaMax microplate reader (Molecular Devices, Germany).

#### Cytokine ELISA

Cytokine production was measured by sandwich ELISA. The Nunc MicroWell flat-bottom 96-well plates (Thermo Fisher Scientific) were coated with primary antibodies (α-IL-4, α-IL-5, α-IL-10, α-IL-9, α-IL-13, α-IFN-γ, α-TNF, and α-TGF-β) and incubated at 4°C, overnight. Coated microplates were blocked with blocking buffer (1× PBS with 2% (m/v) BSA) for 3 hours at 37°C. Appropriately diluted standards and serum and tissue homogenates were added to the designated wells on the microplate and were incubated at 4°C, overnight. Specific biotinylated secondary antibodies (depending on primary Ab) were added and incubated for 2 hours at 37°C. Microplates were developed by adding streptavidin conjugated to either alkaline phosphatase (AP) or horse radish peroxidase (HRP) and incubated at 37°C for 1 hour. An appropriate substrate (3,3’5,5’-Tetramethylbenzidine (TMB) Microwell Peroxidase Substrate (KPL, Gaithersburg, MD, US) for HRP-conjugated secondary antibody or 4-nitrophenol (Sigma) for AP-conjugated secondary antibody) was added to the microplate and incubated for 10 minutes at 37°C. The absorbance was read at 405nm for AP or 450nm for HRP using VersaMax microplate reader (Molecular Devices, Germany).

### Tissue homogenates

Infected liver samples were homogenized in extraction buffer (1× PBS with 2µg protease inhibitor [Sigma-Aldrich, St. Louis, MO, US] and 0.1% Tween 20 [Merck]), spun and supernatants were collected for cytokine detection by sandwich ELISA. Protein concentration was measured using the Pierce BCA Protein Assay Kit (Thermo Fisher Scientific) following the manufacturer’s instructions.

### Liver enzymes

The concentrations of alanine transaminase (ALT) and aspartate transaminase (AST) was determined by diluting the serum 1:10 in 0.9% (m/v) NaCl. The diluted serum was sent to National Health Laboratory Service (NHLS) at Groote Schuur Academic Hospital in Cape Town for analysis.

### Assessment of collagen content in naïve and infected tissue

Hydroxyproline (OH) content was quantified using a modified protocol (Bergman et al., 1963). Briefly, liver samples were weighed and hydrolysed overnight at 110°C in 6M HCl. Hydrolyzed liver samples were diluted with double distilled water (ddH_2_O) and filtered using Whatmann No.1 filter paper. The samples were neutralized with 1% (m/v) phenolphthalein and titrated with 10N NaOH and 3N HCl. Aliquots were mixed with isopropanol and Chloramine-T/Citrate buffer solution (Sigma) was added to the samples. Erlich’s reagent was added, and absorbance was read at 558nm (excitation) and 570nm (emission) using the VersaMax microplate reader (Molecular devices, USA). Hydroxyproline concentrations were determined using 4-hydroxy-L-proline (Calbiochem, San Diego, CA, US) as a standard and data was expressed as µmoles hydroxyproline per weight of tissue that contained 10^4^ eggs.

### *Ex vivo* restimulation of cells

Single cell suspensions were prepared from hepatic lymph node (hLN) harvested from infected mice. Single cell suspensions (1 × 10^6^ cells/ml) were seeded on a 96-well plate coated with α-CD3 (20μg/ml) and incubated in a humidified atmosphere containing 5% CO_2_ at 37°C, and supernatants were collected after 72 hours. Supernatants were collected and cytokines were detected using sandwich ELISA described above.

### cDNA synthesis and quantitative Real Time-PCR

Ribonucleic acid (RNA) was extracted from the liver of infected mice using the Qiagen RNeasy mini kit following the manufacturer’s instructions. RNA was reverse-transcribed into cDNA using random hexamer and anchored-oligo primers. CysLTR1 cDNA was amplified using the following primers: *mCysLTR1* Forward – 5’ - CAA CGA ACT ATC CAC CTT CAC C - ‘3, *mCysLTR1* Reverse – 5’ - AGC CTT CTC CTA AAG TTT CCA C - ‘3. Data were normalized using the hypoxanthine phosphoribosyl transferase (*HPRT*) housekeeping gene using *HPRT* Forward – 5’ - GTT GGA TAT GCC CTT GAC - ‘3 and *HPRT* reverse – 5’ - AGG ACT AGA ACA CCT GCT - ‘3.

### Quantification of egg burden in tissue

Infected liver tissues were weighed and hydrolysed in 5% (m/v) KOH at 37°C overnight. Hydrolyzed tissues were spun down at 2000rpm for 10 minutes and excess supernatant was removed. Eggs in the supernatant were enumerated under inverted light microscope and number of eggs were normalized to tissue weight.

### Flow cytometry and intracellular cytokine staining

Single cell suspensions were prepared from lymph nodes, lungs and liver obtained from either naïve or infected mice and the cells were stained with a cocktail of the following antibodies: CD44, Siglec-F, CD62L, CD11c, F4/80, T1/ST2, CD11b, CD4, CD3, CD8, Ly6G purchased from BD Biosciences (Franklin Lakes, NJ, US) and Biolegend (San Diego, CA, USA). For intracellular cytokine staining, cells were restimulated with a cocktail of 50 ng/ml Phorbol 12-Myristate 13-Acetate (PMA), 250 ng/ml ionomycin and 200 µM monensin for 6 hours at 37°C. After fixation, the cells were permeabilized using the transcription factor buffer set (BD Bioscience). All the antibodies (IL-4, IL-5, IL-9, IL-10, IL-13, IFN-γ, Foxp3, Gata3) were purchased from BD Pharmingen (San Diego, CA, USA) except were noted otherwise. The cells were acquired on a BD LSR Fortessa machine (BD Immunocytometry system, San Jose, CA, USA) and data were analysed using FlowJo software (Treestar, Ashland, OR, USA).

### Statistics

All data are presented as means ± standard error of the mean (SEM), and the *p*-value was determined using Student’s *t* test. Statistical analysis for the present study was conducted using GraphPad Prism 6.0 software (http://www.prismsoftware.com).

## Results

### Generation and characterization of cysLTR1^-/-^ BALB/c mice

CysLTR1 deficient mice were generated by homologous recombination by cleaving out a portion of *cysLTR1* and inserting neomycin cassette (**Supplementary Figures S1A, B**). Homozygous C57BL/6 cysLTR1^-/-^ mice were intercrossed with BALB/c mice to F_10_ generation to generate a stable cysLTR1^-/-^ mice on BALB/c background (**Supplementary Figure S1B**). The deletion of *cysLTR1* was confirmed by conventional polymerase chain reaction (PCR), an expected band size for the disruption of cysLTR1 was 333bp, no disruption at 284bp and heterozygous mice were indicated by double bands at 284bp and 333bp (**Supplementary Figure S1C**). To further confirm the deletion of *cysLTR1*, quantitative real-time PCR (qRT-PCR) was conducted on genomic DNA extracted from spleen, mesenteric lymph node (MLN), lung, liver, and gut of naïve cysLTR1^-/-^ mice and normalized to hypoxanthine-guanine phosphoribosyl transferase (*HRPT*). As expected, there was no expression of *cysLTR1* in cysLTR1^-/-^ mice compared to littermate control mice (**Supplementary Figure S1D**). These results revealed that *cysLTR1* was successfully deleted across tissues in cysLTR1^-/-^ BALB/c mice and confirmed that cysLTR1 BALB/c mice were a global knockout.

### Disruption of cysLTR1 does not alter gross pathology, tissue cellularity however there is an expansion of CD4^+^ and CD8^+^ Tcm in secondary lymphoid tissues under steady state conditions

**Figure 1:**
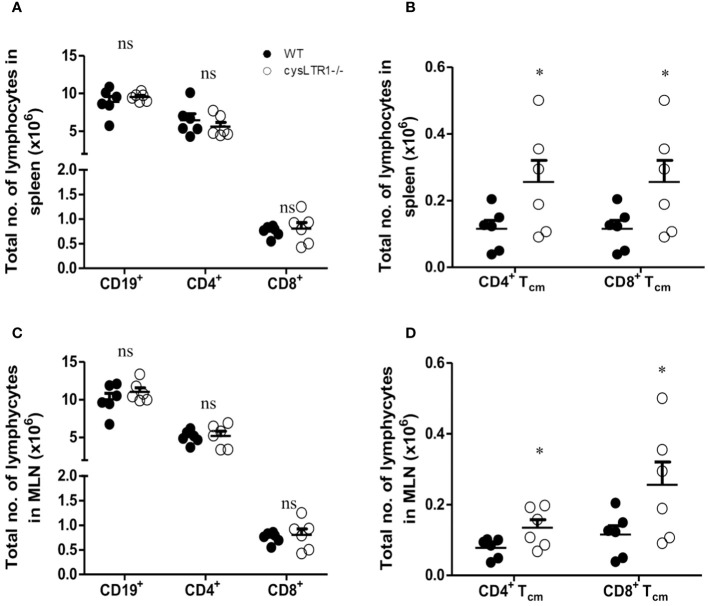
Disruption of cysLTR1 does not alter immune cell composition but there is expansion of CD4 T cm and CD8 Tcm in secondary lymphoid tissues. **(A)** Total number of splenic immune cell populations (CD4, CD8, CD19 B cells). **(B)** Total number of CD4 T central memory (Tcm) and CD8 Tcm in the spleen. **(C)** Total number of MLN immune cell populations (CD4, CD8, CD19 B cells). **(D)** Total number of CD4 Tcm and CD8 Tcm in the MLN. Data are representative of two independent experiments. n=6 - 8 mice. *p<0.05 by unpaired Student’s t-test. ns, statistically not significant.

To determine the impact of deleting *cysLTR1* at homeostasis in naïve mice, we assessed body and vital organs weights, as well as tissue cellularity. There were no apparent differences in body weight (**Supplementary Figure S2A**), vital organ weights (**Supplementary Figure S2B**) and tissue cellularity (**Supplementary Figure S2C**) between cysLTR1^-/-^ and littermate control mice. Furthermore, cysLTR1^-/-^ mice had comparable myeloid cell compartments in the spleen, MLN, lung, liver, and gut (**Supplementary Figures S2D–H**). Additionally, cysLTR1 expression also did not appear to affect the lymphocyte compartments in the thymus (**Supplementary Figure S2I**) and proportions of CD19^+^ B cells, CD4^+^ and CD8^+^ T cells in the spleen (**Figure 1A**), MLN (**Figure 1C**), lung, liver and gut (**Supplementary Figures S2J–L**); however, there was expansion of CD4^+^ and CD8^+^ central memory T cells (CD62L^+^CD44^+^) in secondary lymphoid organs (spleen and MLN) in mice deficient of cysLTR1 in comparison with littermate control mice (**Figures 1B, D**), respectively. Collectively, these data suggested that the lack of cysLTR1 expression in secondary lymphoid tissue results in expansion of CD4 and CD8 Tcm in naïve specific pathogen-free mice without the exposure to foreign antigen. Overall, cysLTR1 BALB/c deficient mice reproduced normally and exhibited no physical abnormalities.

### CysLTR1 deletion leads to reduced liver granulomatous inflammation during chronic schistosomiasis in mice

**Figure 2:**
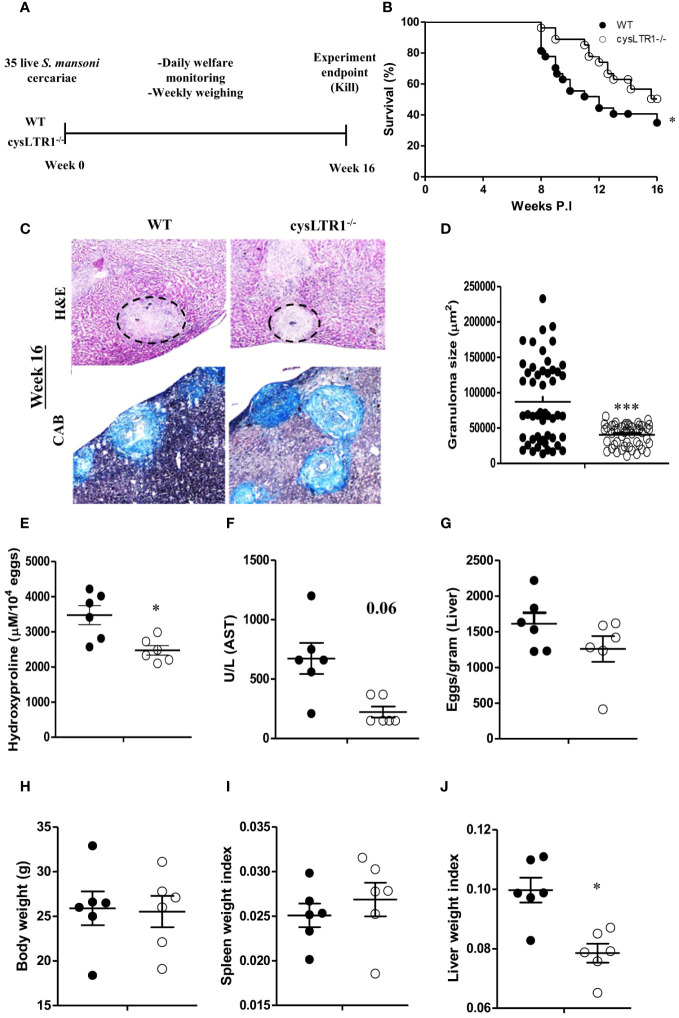
Deletion of cysLTR1 leads to amelioration of granulomatous inflammation during chronic schistosomiasis. CysLTR1 deficient mice and littermate control mice were infected with 35 live *S. mansoni* cercariae, killed at 16 weeks post infection respectively and the liver was harvested. **(A)** Experimental layout. **(B)** Survival curve of *S. mansoni* infected cysLTR1 and control mice. Survival curves were compared using log-rank test. cysLTR1 mice were infected with *S. mansoni* cercariae and analyzed 16 weeks post infection. **(C)** Histological examination of H&E and CAB-stained liver sections. **(D)** Granuloma area was measured using computerized morphometric analysis (NIS Elements, Nikon) by measuring 20 - 25 granulomas per mouse. **(E)** Liver fibrosis determined by assaying hydroxyproline concentration normalized to tissue eggs. **(F)** Hepatocellular damage indicated by serum aspartate transaminase (AST) concentration. **(G)** Egg burden in S-mansoni infected liver. **(H)** Body weight. **(I)** Spleen weight index (as a ratio of total body weights). **(J)** Liver weight index. Data are representative of two independent experiments. n= 6 - 10 mice. *p<0.05 and ***p<0.001 vs wild type mice using unpaired Student’s t-test.

Cysteinyl leukotrienes are essential for activation of Th2 immune responses during allergic asthma and helminth infections (23, 24, 38). To determine whether signalling via cysLTR1 is required for host survival during chronic schistosomiasis, we percutaneously infected cysLTR1^-/-^ mice and littermate control mice with 35 live *S. mansoni* cercariae (**Figure 2A**) and disease outcome was monitored until the attainment of pre-defined humane endpoint (persistent bloody diarrhoea, severe lethargy, and weight loss of 20% or more) (**Supplementary Figure S3A**). About 20% of wildtype mice succumbed to infection while less than 5% of cysLTR1^-/-^ mice had died at 8 weeks pi (**Figure 2B**). More than 55% of wildtype mice died by 12 weeks pi compared to cysLTR1 deficient mice (**Figure 2B**). *In vivo* expression of *cysLTR1* was measured in the liver of naïve and *S. mansoni*-infected BALB/c mice using qPCR and was found to be reduced at both the early and late phases of schistosomiasis (**Supplementary Figure S3B**). Despite the better resilience to the disease, we observed that cysLTR1 deficient mice developed wasting disease and lost 9% of body weight by 10 weeks pi compared to littermate control mice (**Supplementary Figure S3C**). Therefore, these data indicated that the absence of cysLTR1 enhanced the survival of mice during chronic schistosomiasis.

We sought to understand whether liver pathology was affected during chronic schistosomiasis in mice deficient of cysLTR1 compared to littermate control mice. Histological analysis revealed that cysLTR1^-/-^ mice developed significantly reduced inflammation as judged by the smaller granuloma size compared to littermate control mice (**Figures 2C, D**). Furthermore, we observed reduced fibrosis indicated by reduced hydroxyproline content (**Figure 2E**) and a trend towards reduced liver injury as indicated by aspartate transaminase (**Figure 2F**) in the liver of cysLTR1^-/-^ mice as compared to littermate control mice. There were no differences in the quantity of parasitic eggs lodged in the liver between the two groups (**Figure 2G**). Although the overall body weight (**Figure 2H**) and spleen sizes remained comparable during chronic schistosomiasis (**Figure 2I**), the size of the liver was significantly smaller, translating into a significantly reduced level of hepatomegaly in cysLTR1^-/-^ mice as compared to wildtype mice (**Figure 2J**). Overall, this data suggests absence of cysLTR1 leads to reduced liver inflammation and damage.

**Figure 3:**
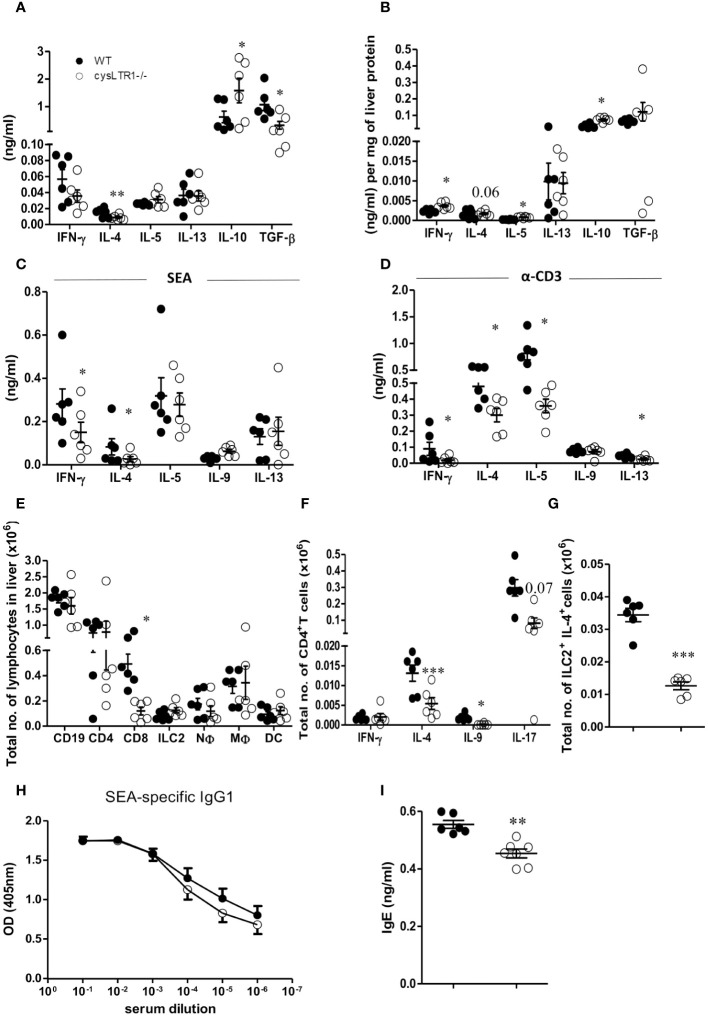
Deletion of cysLTR1 leads to reduced Th2 immune response during chronic schistosomiasis. CysLTR1 deficient mice and littermate control mice were infected with 35 live *S. mansoni* cercariae, killed at 16 weeks post infection and the serum, hLN and liver were harvested. **(A)** Cytokine production in the serum. Livers from infected mice were homogenized and levels of the indicated **(B)** cytokines were detected by ELISA and normalized to mg of liver tissue at week 16 post infection. Total hLN cells were stimulated with **(C)** SEA and **(D)** αCD3. **(E)** Representative total number of CD4^+^ intra-epithelial lymphocytes (IEL), CD8^+^ IEL, CD4^+^ CD8^+^ IEL, CD8^+^ dendritic cells, neutrophils (CD11b^+^Ly6G^+^), macrophages (CD11b^+^F4/80^+^), and eosinophils (CD11b^+^SiglecF^+^). **(F)** Total number of IFN-γ, IL-4, IL-9, and IL-17-expressing CD4^+^ T cells. **(G)** Total number of IL-4 expressing ILC2 cells in the liver. **(H)** SEA-specific IgG1 antibody titre. **(I)** Total IgE antibody titre. Data are representative of two independent experiments. n=6 - 10 mice. *p<0.05, **p<0.01 and ***p<0.001 by unpaired Student’s t-test.

To further investigate the mechanism associated with reduced liver pathology in infected cysLTR1^-/-^ mice during chronic schistosomiasis, serum cytokine and immune responses were assessed in the liver and hepatic lymph nodes (hLN). There was a significant reduction of type 2 (IL-4) and regulatory (TGF-β) cytokines and upregulation of IL-10 in cysLTR1^-/-^ mice as compared to controls in the serum (**Figure 3A**). The reduced IL-4 production and elevated levels of IL-10 were consistent in the liver homogenates in the chronic phase in the absence of cysLTR1 (**Figure 3B**). Further analysis of cytokine immune response in liver homogenates revealed increased levels of IFN-γ in liver at the chronic phase in cysLTR1^-/-^ mice compared with littermate control mice (**Figure 3B**). *In vitro* stimulation of total hLN cells with SEA or α-CD3 indicated a consistent reduced IL-4 and IFN-y production (**Figures 3C, D**) in mice lacking cysLTR1 compared to littermate control mice. We also noted reduced type 2 cytokines IL-5 and IL-13 in the hLN restimulated with α-CD3 in cysLTR1^-/-^ mice as compared to littermate control mice (**Figure 3D**). To assess the immune cells, a gating strategy (**Supplementary Figure S5**) was used. The infiltration of immune cells remained comparable at week 16 with the exception of reduced CD8^+^T cells in the absence of cysLTR1 compared to littermate control mice (**Figure 3E**). Consistent with phenotype in the serum, liver homogenates and *in-vitro* restimulation of hLN cells with SEA or αCD3, we observed reduced intracellular production of IL-4 by CD4^+^ T (**Figure 3F**). We also noted reduced levels of IL-9 and IL-17 during the late phase of the disease, as well as intracellular expression of IL-4 expression by ILC2 (**Figure 3G**) in cysLTR1^-/-^ mice compared to littermate control mice. Assessment of type 2 SEA-specific IgG1 (**Figure 3H**) revealed a comparable measure between two groups; however, total IgE titre (**Figure 3I**) was significantly reduced in cysLTR1^-/-^ mice as compared to the littermate control mice. Taken together, these data demonstrated that cysLTR1 is necessary for mounting sufficient type 2 immune responses during chronic schistosomiasis in mice.

### Inhibition of cysLTR1 with montelukast together with praziquantel results in the expansion of CD4^+^Foxp3^+^ T cells during chronic schistosomiasis

**Figure 4:**
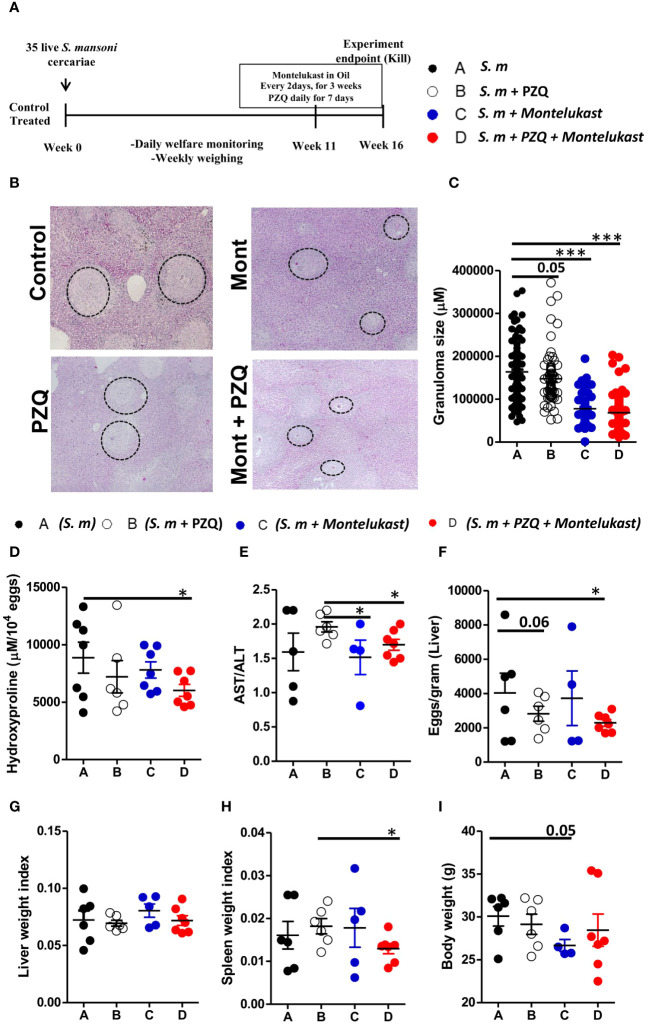
Pharmacological blockade of cysLTR1 led to reduced liver pathology during chronic schistosomiasis. Four groups of wild type mice were infected with 35 live *S. mansoni* cercariae and at 11 weeks pi, one group was treated with Praziquantel only once daily for 7 days, Montelukast only once every other day for 3 weeks, a combination of Praziquantel and Montelukast therapy and one group was given a mock treatment and kept as a control. Animals were killed at week 16 pi and the liver and serum were harvested. **(A)** Experimental layout. **(B)** Histological examination of H&E-stained liver sections. **(C)** Granuloma area was measured using computerized morphometric analysis (NIS Elements, Nikon) by measuring 20 - 25 granulomas per mouse. **(D)** Liver fibrosis determined by assaying hydroxyproline concentration normalized to tissue eggs. **(E)** Hepatocellular damage indicated by serum aspartate transaminase (AST)/alanine transaminase (ALT) ratio. **(F)** Parasite egg burden in liver. **(G)** Liver weight index. **(H)** Spleen weight index. **(I)** Body weight. Data are representative of two independent experiments. n= 4 - 7 mice. *p<0.05 and ***p<0.001 by unpaired Student’s t-test.

Next, we determined whether inhibition of cysLTR1 alone or in combination with praziquantel (PZQ) could ameliorate liver pathology during chronic phase of schistosomiasis. We infected four groups of wildtype mice with 35 live *S. mansoni* (low dose) cercariae, and at week 11, one group was treated with PZQ for one week; one group was treated with montelukast once every two days for 3 weeks; one group was treated with montelukast in combination with PZQ while the control group was mock treated (**Figure 4A**). All four groups of mice were killed at week 16 pi. We observed reduced liver granulomatous inflammation in the mice treated with montelukast alone and in combination with PZQ (**Figures 4B, C**). We also noted a trend towards reduced granuloma size in PZQ-treated mice; however, the differences were not significant (**Figures 4B, C**). Further analysis revealed reduced liver hydroxyproline quantity in mice treated with combination therapy as compared to control group (**Figure 4D**). Moreover, we observed that mice that were treated with montelukast alone or in combination with PZQ displayed significantly reduced ratio of AST/ALT as compared to control mice (**Figure 4E**), indicating reduced hepatoxicity following these treatments. We observed no difference in liver egg burdens between the infected mock control mice and mice treated PZQ or montelukast alone, while there was a reduced liver egg burden in mice treated with a combination of montelukast and PZQ compared to mock control mice (**Figure 4F**). There was no difference in liver weight between all the infected groups (**Figure 4G**); however, we did observe a significantly reduced spleen weight in mice treated with a combination of montelukast and PZQ compared to mice treated with PZQ alone (**Figure 4H**), indicating that the combined therapy reduced the spleen pathology compared to the single PZQ therapy alone. Mice treated with montelukast showed a trend towards reduced body weight (**Figure 4I**), which was not evident in the rest of the infected groups. Overall, these data suggests that combined therapy with montelukast and PZQ drives reduced egg-driven fibrogranulomatous inflammation in the liver during chronic schistosomiasis in mice.

**Figure 5:**
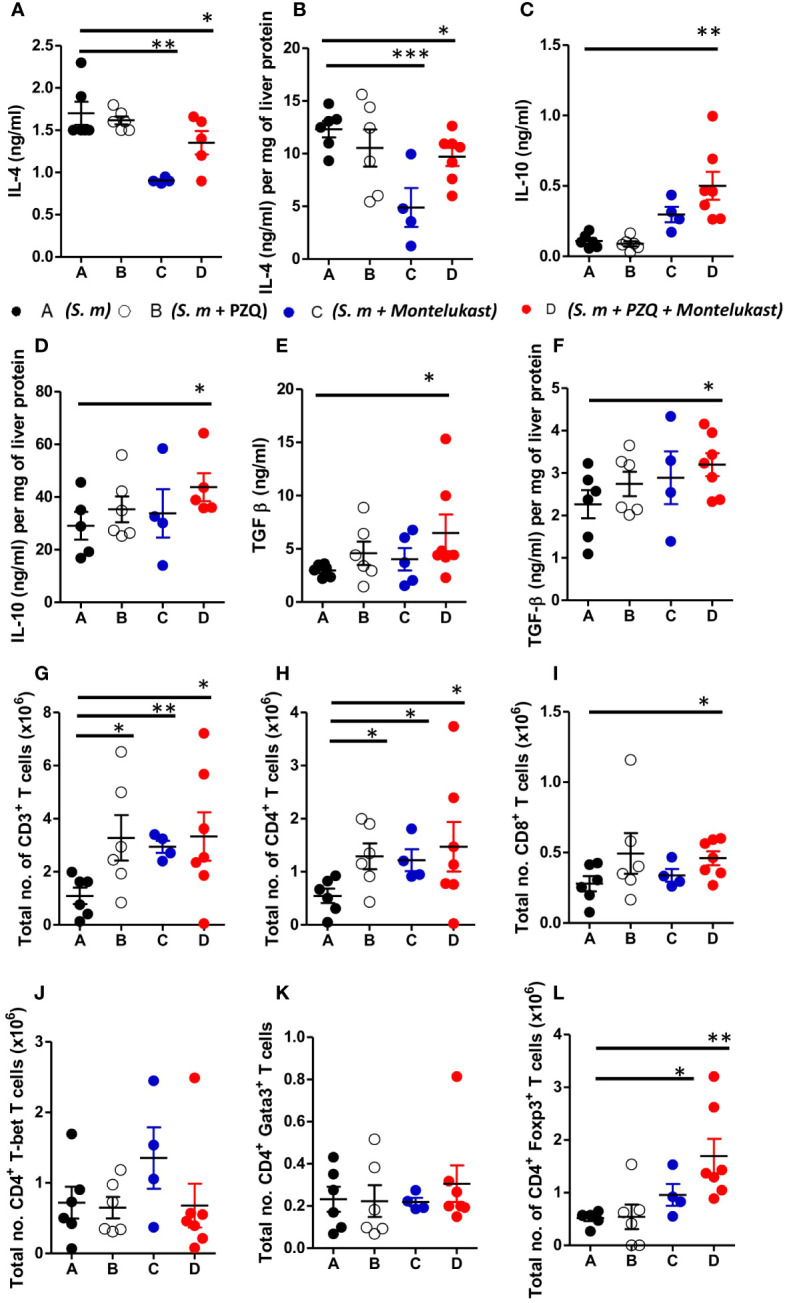
Inhibition of cysLTR1 in combination PZQ results in expansion of CD4^+^Foxp3^+^ T cells during chronic schistosomiasis. **(A–F)** Cytokine production in the serum and liver homogenates (Livers from infected mice were homogenized and levels of the indicated cytokines were detected by ELISA and normalized to mg of liver tissue at week 16 post infection). Representative total number of CD3^+^ lymphocytes **(G)**, CD4^+^ lymphocytes **(H)** and CD8^+^ lymphocytes **(I)**. Total number of T-bet **(J)**, Gata3 **(K)** and Foxp3 **(L)** expressing CD4^+^ T cells. Data are representative of two independent experiments. n= 4-7 mice. *p<0.05, **p<0.01 and ***p<0.001 by unpaired Student’s t-test.

To understand the cellular mechanism of the combined therapy during chronic schistosomiasis, we examined the immune responses in the serum and liver of the infected animals. Treatment with montelukast alone and in combination with PZQ led to reduced IL-4 cytokine production in serum and liver homogenates as compared to control mice (**Figures 5A, B**). Furthermore, combination therapy also resulted in heightened production of IL-10 and TGF-β compared to infected mock treated mice (**Figures 5C–F**). There was an upregulation of IL-10 (**Figures 5C, D**) and TGF-β (**Figures 5E, F**) in the serum and liver homogenates following combined therapy as compared to the control mice. We also observed an overall expansion of total number of CD3^+^ T cells (**Figure 5G**), CD4^+^ T cells (**Figure 5H**) and CD8^+^ T cells (**Figure 5I**) in the infected liver of mice given a single or combination therapy as compared to control groups during chronic schistosomiasis. Although there were no significant differences in the total CD4^+^ T cells expressing T-bet (**Figure 5J**) and Gata3 T cells (**Figure 5K**) between all groups, we noted a significantly increased number of CD4^+^ T cells expressing Foxp3 (**Figure 5L**) in mice that were treated with montelukast alone or with combination therapy compared to infected mock treated control mice. Infected mice treated with montelukast alone or in combination with PZQ displayed significantly reduced total IgE titres compared to infected mock treated control mice (**Supplementary Figure S4A**) while SEA-specific IgG1 titres (**Supplementary Figure S4B**) remained comparable in all the different groups. We also noted an upregulation of type 1 antigen specific IgG2a (**Supplementary Figure S4C**) antibody responses in mice that received a combination therapy compared to infected mock treated mice during chronic schistosomiasis, while no differences were noted in SEA-specific IgG2b antibody titres between all groups (**Supplementary Figure S4D**). Collectively, our data suggests that the combination therapy with montelukast and PZQ leads to reduced type 2 responses and an expansion of regulatory T cells which could be associated with control of fibrogranulomatous inflammation in liver of the chronically infected mice.

### Deletion of cysLTR1 ameliorates hepatic granulomatous inflammation during acute phase of schistosomiasis in mice

**Figure 6:**
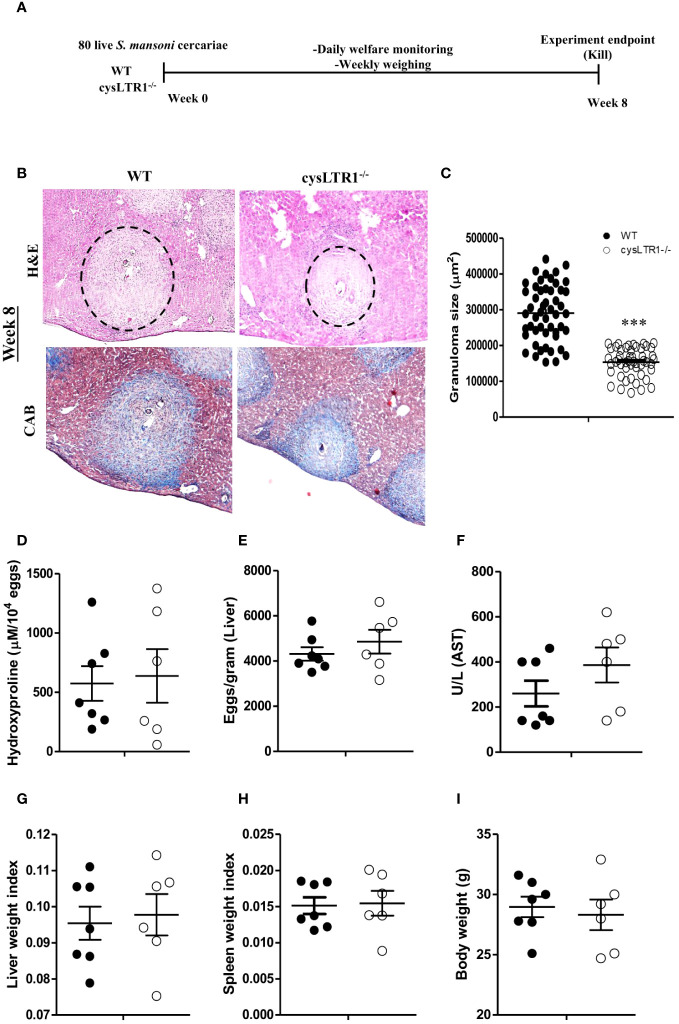
Deletion of cysLTR1 leads to amelioration of granulomatous inflammation during acute schistosomiasis. CysLTR1 deficient mice and littermate control mice were infected with 80 live *S. mansoni* cercariae, killed at 8-weeks post infection and the liver was harvested. **(A)** Experimental layout. **(B)** Histological examination of H&E and CAB-stained liver sections. **(C)** Granuloma area was measured using computerized morphometric analysis (NIS Elements, Nikon) by measuring 20 - 25 granulomas per mouse. **(D)** Liver fibrosis determined by assaying hydroxyproline concentration normalized to tissue eggs. **(E)** Egg burden in S-mansoni infected liver. **(F)** Hepatocellular damage indicated by serum aspartate transaminase (AST) concentration. **(G)** Liver weight. **(H)** Spleen weight index. **(I)** Body weight. Data are representative of two independent experiments. n= 6 - 7 mice. ***p<0.001 vs wild type mice using unpaired Student’s t-test.

Seeing that the absence and the inhibition of cysLTR1 led to reduce in liver inflammation during chronic schistosomiasis, we questioned whether the same phenotype was true during acute schistosomiasis. We percutaneously infected cysLTR1^-/-^ mice and littermate control mice with 80 live *S. mansoni* cercariae and assessed the mice at 8 weeks pi (**Figure 6A**). Histological analysis revealed that mice lacking cysLTR1 exhibited 2-fold smaller hepatic granuloma size as compared to the littermate control mice (**Figures 6B, C**). We noted no differences in the hepatic collagen content (**Figure 6D**), parasitic egg burden (**Figure 6E**) and liver enzymes (**Figure 6F**) between the two groups of mice. In addition, no differences were observed in the liver weight (**Figure 6G**), spleen weight (**Figure 6H**) and overall body weight (**Figure 6I**) between the two mice groups. We concluded that although cysLTR1 is crucial for hepatic granuloma formation, it remains dispensable for other tissue pathology during acute schistosomiasis.

## Discussion

Understanding the essential role of cysLTs signalling through cysLTR1 on immune system function has been primarily focused on asthma and allergic rhinitis. The present study aimed at evaluating the role of cysLTR1 signalling during experimental schistosomiasis in murine model. Mice lacking cysLTR1 survived *S. mansoni* infection better than littermate control mice. Previous studies have reported that other leukotrienes are crucial for disease control. For instance, a study by Tristão et al. reported that mice deficient of 5-lipoxygenase (5-LO) failed to control *Paracoccidiodes brasiliensis*, a fungal infection (39). Furthermore, mice lacking 5-LO exhibited reduced inflammation, which resulted in protection to *Trypanosoma cruzi* (40), *Mycobacterium tuberculosis* (41) and *S. mansoni* (32). In contrast, mice deficient of 5-LO displayed increased susceptibility to *Toxoplasma gondii* infection (42). A study by Hohmann et al., reported that mice lacking 5-LO had reduced lethality rates compared to littermate control following acetaminophen-induced liver injury (43). The higher susceptibility in littermate control mice coincides with the higher degree of liver damage, as revealed by liver histopathology analysis. Moreover, mice lacking 5-LO displayed reduced liver pathology that was indicated by significantly reduced granuloma size during *Brucella abortus* (*B. abortus*) infection in mice (44). Furthermore, 5-LO deficient mice displayed increased production of proinflammatory cytokines following *B. abortus* infection (44). Indicating the differential roles of leukotrienes have on different disease models. Disruption of cysLTR1 in mice resulted in overall reduced inflammation at weeks 8 and 16 pi as judged by reduced granuloma size in the liver. The collagen content and liver enzymes were reduced at the late phase of infection, an indication that cysLTR1 drives immunopathology during chronic schistosomiasis.

The improved resilience to schistosomiasis during chronic phase of the disease was further associated with impaired IL-4 production. IL-4 contributes to the development of type 2 CD8^+^ T cells (45). It had been suggested that CD8^+^ T cells act as a suppressor of Th2 cell function and thereby regulate granulomatous inflammation (45). Previous studies have shown that protection against schistosomiasis is IL-4 dependent (27, 33, 46–50) resulting in the activation of Th2 immune response. Hoffman et al., demonstrated that the lack of IL-4 in mice led to Th1-like proinflammatory response that leads to severe form of disease and mice quickly succumb to schistosomiasis (25). Although there was impaired production of IL-4 in the absence of cysLTR1, this did not render mice susceptible to chronic schistosomiasis. A study in our lab also revealed that the removal of IL-4Rα (IL-4 and IL-13 receptor binding modulator) during the chronic phase of schistosomiasis resulted in the amelioration of liver granulomatous inflammatory pathology and reduced liver fibrosis, which was supported by impaired Th2 responses and heightened frequencies of Foxp3^+^ T and CD1d^hi^ CD5^+^ B regulatory cells (33). The reduction of IL-4 and an increase of IL-10 is a good profile to help the host cope during the transition from the peak of the acute response to the establishment of chronicity, creating a balanced cytokine profile between IL-4 and IL-10. IL-10 plays an important regulatory role in many infections and inflammatory diseases (51–55), is capable of reducing inflammation and has been shown to offer protection to severe liver damage caused by excessive tissue injury (56). This is of major importance because studies conducted in humans have demonstrated the importance of IL-10 in regulating morbidity during schistosomiasis (26, 57, 58).

Next, we evaluated how the pharmacological inhibition of cysLTR1 alone or in combination with PZQ influenced liver inflammation during chronic schistosomiasis. A study by Pu et al. showed that blocking cysLTR1 with montelukast ameliorated acetaminophen (APAP)-induced acute hepatic injury as indicated by reduced serum ALT and AST levels, reduced necrosis area and reduced inflammatory cytokine gene expression (59). Similarly, inhibition of COX-2, another enzyme of the arachidonic acid pathway, led to the amelioration of liver inflammation during *Schistosoma japonica* infection (60). These studies align with our findings where we observed reduced inflammation as judged by notable reduction in the size of granuloma, as well as reduced liver damage as judged by the AST/ALT levels in the serum upon inhibition of cysLTR1. In other studies, treatment with Montelukast led to reduced lung destruction and inflammatory cytokines during smoke-induced lung injury (61, 62). Similar to our findings, Ikeno and colleagues also revealed interesting correlation between liver fibrosis and T regs. They noted that hepatic Treg cells play a crucial role in preventing liver pathology by subduing inflammatory cellular immunity that contribute to liver damage and fibrosis (63). In agreement with the above observations and our study, Haack et al. demonstrated that Tregs reduced the severity of the pathology of the infected liver during hepatitis in mouse model (64). Furthermore, our findings highlighting the upregulation of regulatory cytokines (IL-10 and TGF- β) together with the expansion of Tregs indicates an elevated control of liver inflammation and fibrosis after the combination therapy in chronically infected mice. Our findings, therefore, raise the intriguing possibility that the combination of montelukast and PZQ could be promising treatment of chronic schistosomiasis.

In conclusion, our study demonstrated positive association of cysLTs signalling through cysLTR1 in the liver pathology of *Schistosoma*-infected mice. The absence of cysLTR1 during *S. mansoni* infection results in reduced liver pathology during different phases of schistosomiasis. We showed that cysLTR1 inhibition with Montelukast ameliorated fibrogranulomatous pathology by blocking Th2 responses and expansion of Treg immune responses, cysLTR1 inhibition could be a possible therapeutic option in combination with Praziquantel for treating fibrogranulomatous pathology during chronic and acute schistosomiasis.

## Data availability statement

The raw data supporting the conclusions of this article will be made available by the authors, without undue reservation.

## Ethics statement

The animal study was approved by UCT Health Sciences Animal Ethics Committee. The study was conducted in accordance with the local legislation and institutional requirements.

## Author contributions

PM: Data curation, Formal analysis, Project administration, Writing – review & editing, Investigation, Methodology, Validation, Writing – original draft. TM: Data curation, Formal analysis, Investigation, Methodology, Writing – review & editing. NA: Data curation, Formal analysis, Investigation, Methodology, Writing – review & editing. HN: Data curation, Formal analysis, Writing – review & editing, Supervision, Writing – original draft. FM: Data curation, Formal analysis, Writing – review & editing, Investigation, Methodology. JN: Formal analysis, Methodology, Writing – review & editing, Conceptualization. FB: Conceptualization, Formal analysis, Writing – review & editing, Data curation, Funding acquisition, Project administration, Resources, Supervision.

## Supplementary material

Supplementary Figure 1 Generation of cysLTR1 deficient BALB/c mice. **(A)** A genomic organization of the mouse cysLTR1 gene (upper), structure of the targeting vector (middle), and organization of the putative recombinant cysLTR1 allele (lower). Exons II-IV are shown as boxes with the coding regions in black. Restriction enzyme sites include BAmHI (B), BgIII (Bg), HindIII (H) and ScaI (S). **(B)** Mouse breeding strategy. cysLTR1 deficient mice were intercrossed for three generations with BALB/c wildtype mouse. **(C)** Genotyping of cysLTR1 deficient mice. DNA was extracted from the tail of naïve cysLTR1 deficient and littermate control mice and PCR was performed. The cysLTR1^+/+^ specific amplicon is 284bp, CysLTR1^-/-^ is 333bp and the cysLTR1^+/-^ is represented by both bands (284bp and 333bp). **(D)** qPCR and normalized to the quantity of hypoxanthine phosphoribosyl transferase (HPRT) which is present in all cells.

Supplementary Figure 2 Absence of cysLTR1 does not alter body weight, tissue weight and cellularity in naïve mice. **(A)** Body weight of naïve sex and age matched mice. **(B)** Organ weights of naïve mice. Total myeloid cell numbers in the **(D)** spleen, **(E)** mesenteric lymph node MLN, **(F)** lung, **(G)** liver, **(H)** gut and total lymphocyte cell numbers of **(I)** thymus, **(J)** lung, **(K)** liver, **(L)** gut of naïve young mice. Data are representative of two independent experiments. n=6 - 8 mice.

Supplementary Figure 3 Absence of cysLTR1 leads to wasting disease during acute schistosomiasis. CysLTR1 deficient mice and wild type mice were infected with 35 live *S. mansoni* cercariae **(A)** Experimental plan, **(B)** cysLTR1 mRNA expression relative to HRPT housekeeping gene by RT-PCR to quantify cysLTR1 mRNA levels on wildtype control mice. **(C)** Kinetics of percentage body weight change over time. Data are representative of two independent experiments. n=10 mice. **p*<0.05 by unpaired Student’s t-test.

Supplementary Figure 4 Inhibition of cysLTR1 leads to expansion of total T cells and B cells, with a reduction of humoral immune response. CysLTR1 deficient mice and wild type mice were infected with 35 live *S. mansoni* cercariae. **(A)** Total IgE antibody titre. SEA-specific IgG1 **(B)**, IgG2a **(C)** and IgG2b **(D)** antibody titre. Data are representative of two independent experiments. n= 4 - 7 mice. **p*<0.05 by unpaired Student’s t-test.

Supplementary Figure 5 Gating strategy. Identification of tissue CD4^+^, CD8^+^ T cells, CD19^+^ B cells, inflammatory macrophages, neutrophils, dendritic cells, eosinophils and ILC2.
