# Supplementary figures and images for: Cysteinyl leukotriene receptor-1 as a potential target for host-directed therapy during chronic schistosomiasis in murine model
Source: Front Immunol. 2024 May 22;15:1279043. doi: 10.3389/fimmu.2024.1279043 (PMC11150569; doi:10.3389/fimmu.2024.1279043)

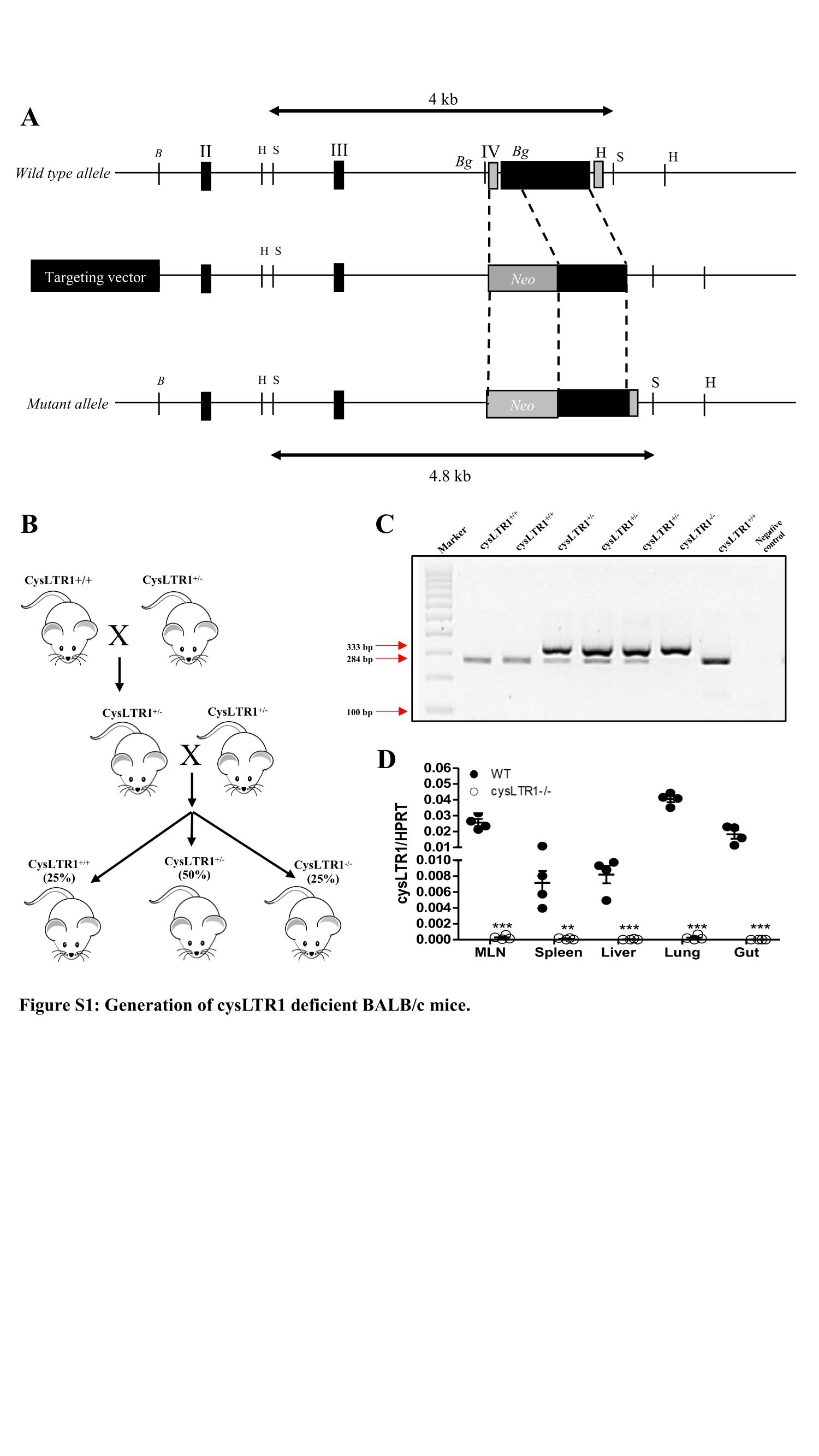

Supplement: Supplementary Figure 1 — Generation of cysLTR1 deficient BALB/c mice. (A) A genomic organization of the mouse cysLTR1 gene (upper), structure of the targeting vector (middle), and organization of the putative recombinant cysLTR1 allele (lower). Exons II-IV are shown as boxes with the coding regions in black. Restriction enzyme sites include BAmHI (B), BgIII (Bg), HindIII (H) and ScaI (S). (B) Mouse breeding strategy. cysLTR1 deficient mice were intercrossed for three generations with BALB/c wildtype mouse. (C) Genotyping of cysLTR1 deficient mice. DNA was extracted from the tail of naïve cysLTR1 deficient and littermate control mice and PCR was performed. The cysLTR1+/+ specific amplicon is 284bp, CysLTR1-/- is 333bp and the cysLTR1+/- is represented by both bands (284bp and 333bp). (D) qPCR and normalized to the quantity of hypoxanthine phosphoribosyl transferase (HPRT) which is present in all cells. [file Image_1.jpeg]

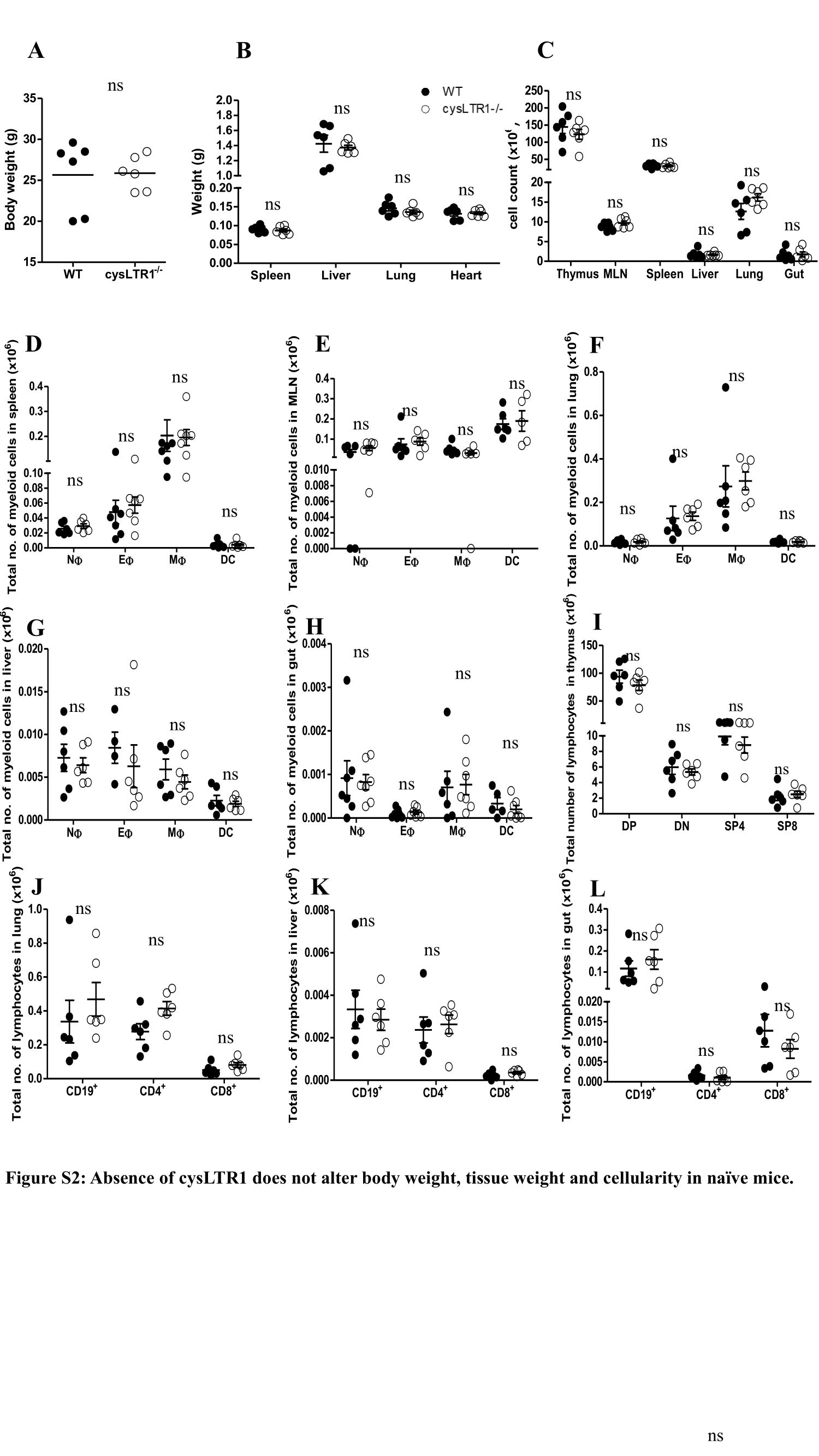

Supplement: Supplementary Figure 2 — Absence of cysLTR1 does not alter body weight, tissue weight and cellularity in naïve mice. (A) Body weight of naïve sex and age matched mice. (B) Organ weights of naïve mice. Total myeloid cell numbers in the (D) spleen, (E) mesenteric lymph node MLN, (F) lung, (G) liver, (H) gut and total lymphocyte cell numbers of (I) thymus, (J) lung, (K) liver, (L) gut of naïve young mice. Data are representative of two independent experiments. n=6 - 8 mice. [file Image_2.jpeg]

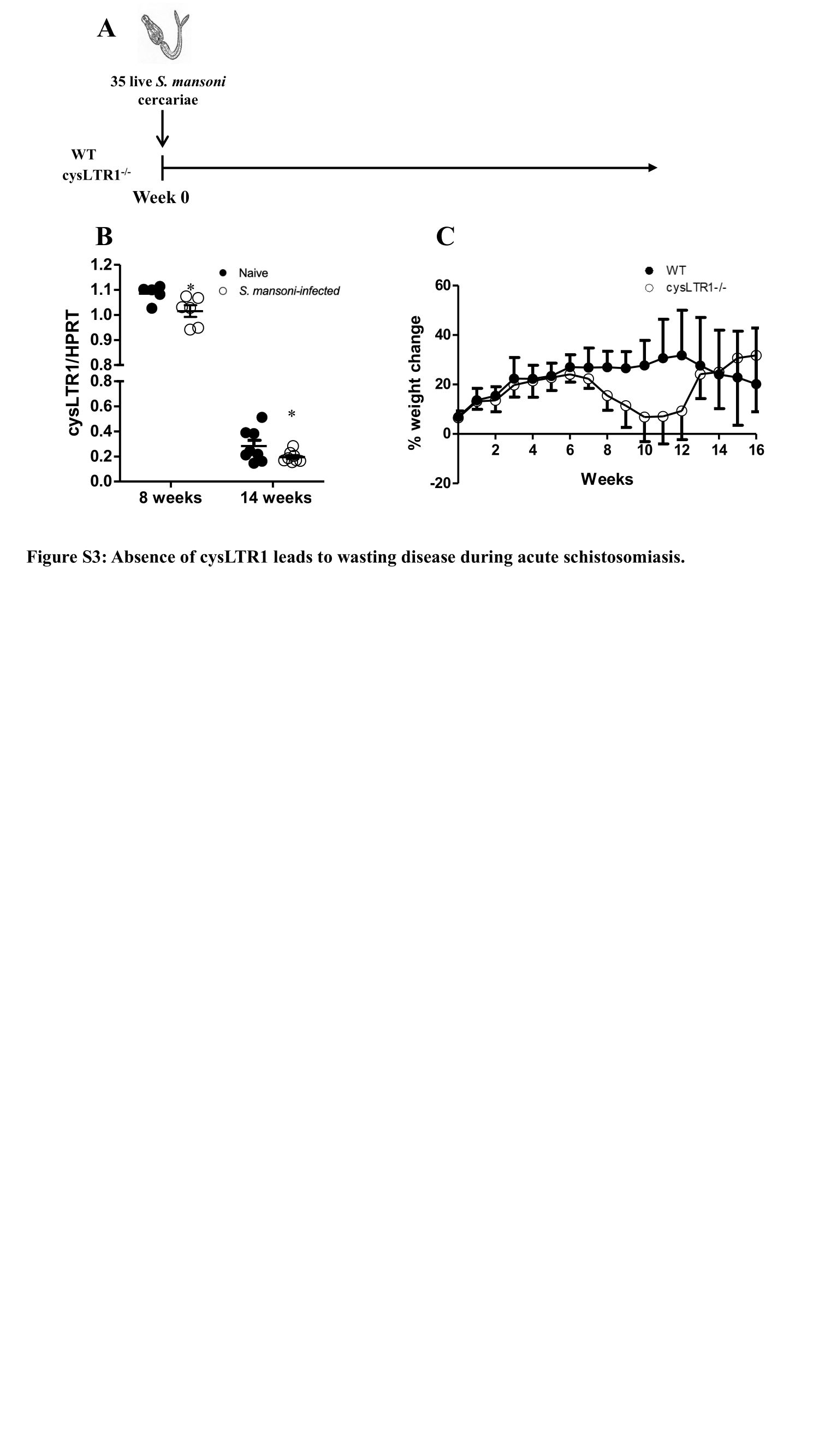

Supplement: Supplementary Figure 3 — Absence of cysLTR1 leads to wasting disease during acute schistosomiasis. CysLTR1 deficient mice and wild type mice were infected with 35 live S. mansoni cercariae (A) Experimental plan, (B) cysLTR1 mRNA expression relative to HRPT housekeeping gene by RT-PCR to quantify cysLTR1 mRNA levels on wildtype control mice. (C) Kinetics of percentage body weight change over time. Data are representative of two independent experiments. n=10 mice. *p<0.05 by unpaired Student’s t-test. [file Image_3.jpeg]

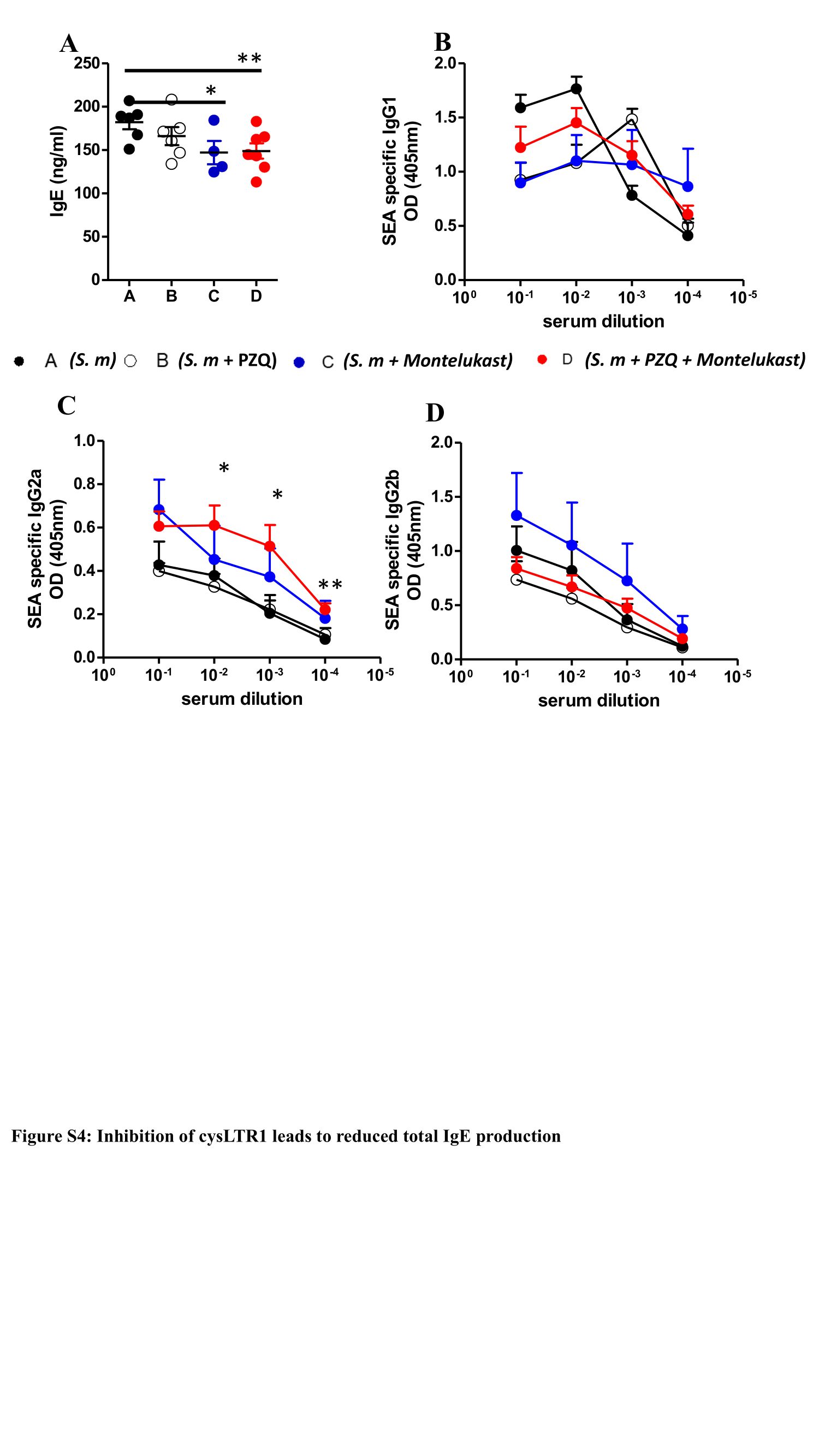

Supplement: Supplementary Figure 4 — Inhibition of cysLTR1 leads to expansion of total T cells and B cells, with a reduction of humoral immune response. CysLTR1 deficient mice and wild type mice were infected with 35 live S. mansoni cercariae. (A) Total IgE antibody titre. SEA-specific IgG1 (B), IgG2a (C) and IgG2b (D) antibody titre. Data are representative of two independent experiments. n= 4 - 7 mice. *p<0.05 by unpaired Student’s t-test. [file Image_4.jpeg]

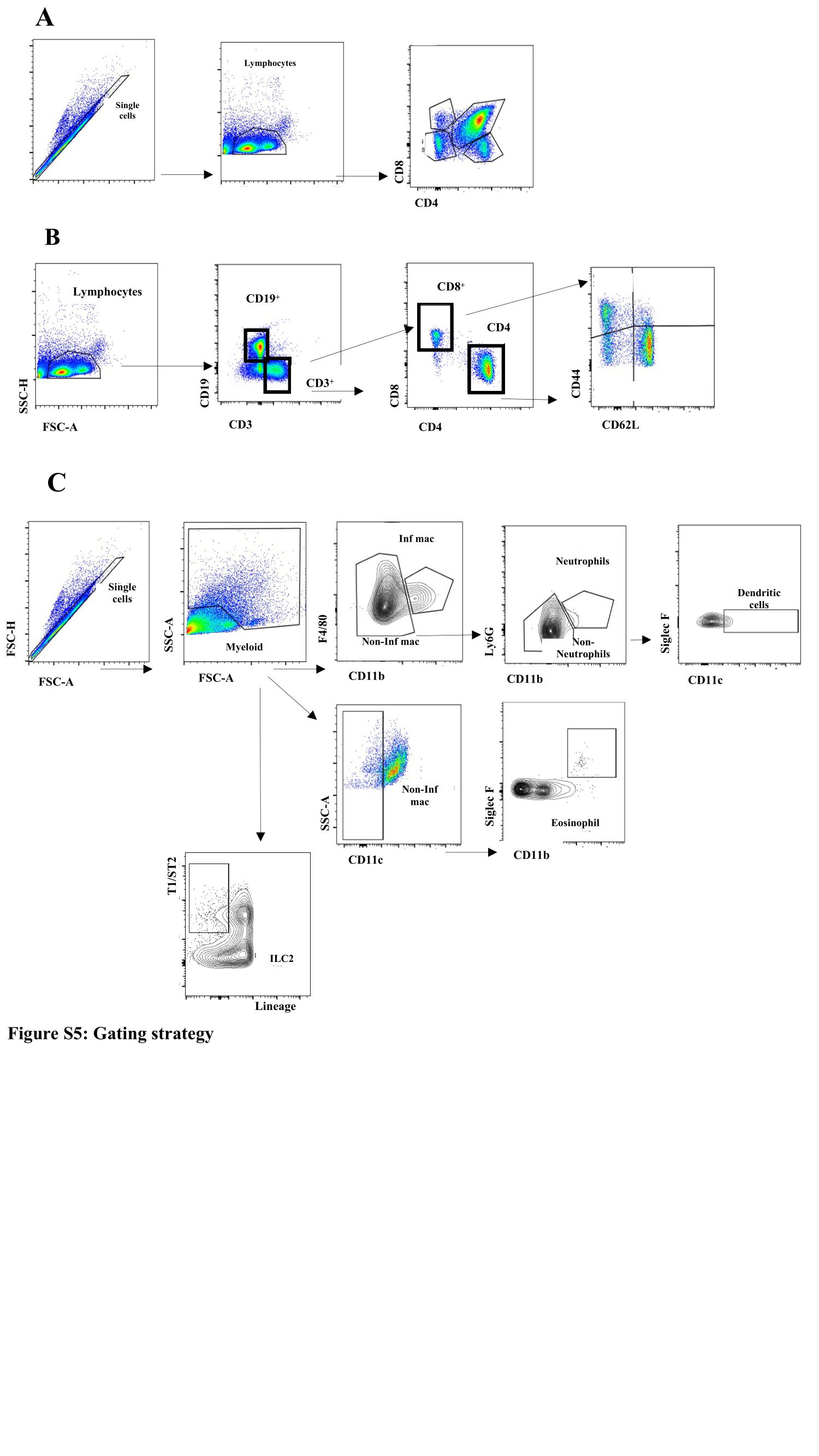

Supplement: Supplementary Figure 5 — Gating strategy. Identification of tissue CD4+, CD8+ T cells, CD19+ B cells, inflammatory macrophages, neutrophils, dendritic cells, eosinophils and ILC2. [file Image_5.jpeg]
